# Supplementary material for: Molecular basis of SAP05-mediated ubiquitin-independent proteasomal degradation of transcription factors
Source: Nat Commun. 2024 Feb 8;15:1170. doi: 10.1038/s41467-024-45521-7 (PMC10850148; doi:10.1038/s41467-024-45521-7)
Supplement: Supplementary file 1 — Supplementary information [file 41467_2024_45521_MOESM1_ESM.pdf]

Supplementary information for

Molecular basis of SAP05-mediated ubiquitin-independent proteasomal degradation of transcription factors

Xiaojie Yan[1,2,#], Xinxin Yuan[1,2,3,#], Jianke Lv[1,4,#], Bing Zhang[1,2,#], Yongle Huang[2], Qianqian Li[1,4], Jinfeng Ma[3], Yanran Li[2], Xiaolu Wang[5], Yao Li[2], Ying Yu[5], Quanyan Liu[3], Tong Liu[6], Wenyi Mi[1,4,*], and Cheng Dong[1,2,3,6*]

*Corresponding author. Email: dongcheng@tmu.edu.cn or wenyi.mi@tmu.edu.cn

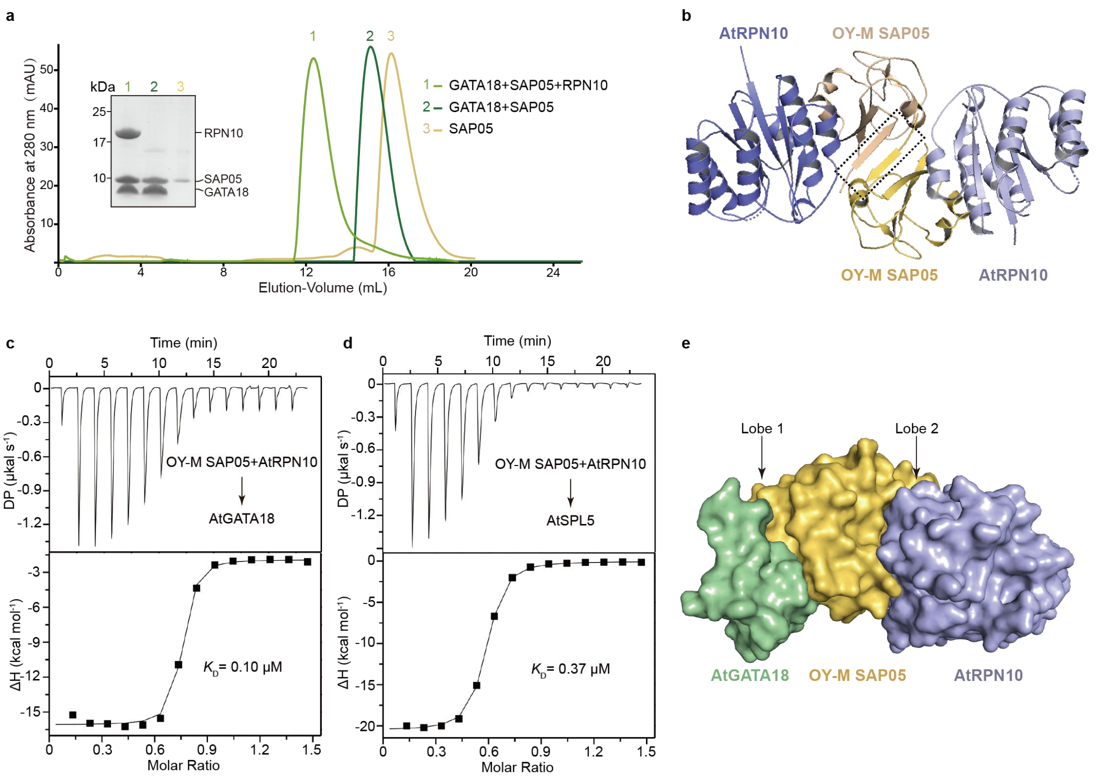

Supplementary Fig. 1 | SAP05 bound to GATA18/SPL5 and RPN10. a, Gel-filtration chromatography profiles of SAP05 alone and in complex with GATA18 and GATA18-RPN10, as analyzed by Superdex 75 Increase 10/300. The left panel shows an SDS-PAGE gel of the peak fractions, stained with Coomassie blue. Source data are provided as a Source Data file. Representative images, n=3. **b,** Crystal structure of OY-M SAP05-AtRPN10 complex in one crystallographic asymmetric unit, where the N-terminus of SAP05 forms a β-strand owing to packing against its counterpart. **c-d,** ITC measurements of binding affinities (K_D) of SAP05-AtRPN10 to AtGATA18 and AtSPL5, respectively. **e,** A surface diagram of the GATA18-SAP05-RPN10 complex, generated by superposing the structure of SAP05 from the single complex GATA18-SAP05 and SAP05-RPN10.

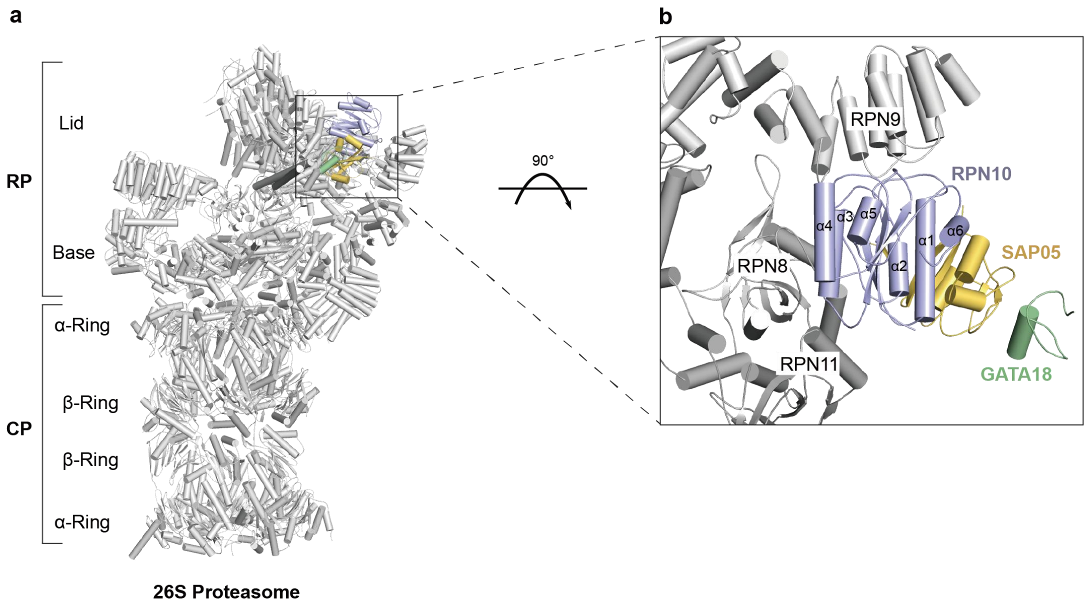

Supplementary Fig. 2 | The binding model of SAP05 bridges GATA18 to the RPN10 in the 26S proteasome. a, Architecture of the human 26S proteasome (PDB: 6MSE), the AtRPN10 in the SAP05-AtRPN10 complex is superimposed on human RPN10 in the 26S proteasome. **b,** Close-up view of RPN10-mediated contacts with the 26S proteasome and SAP05.

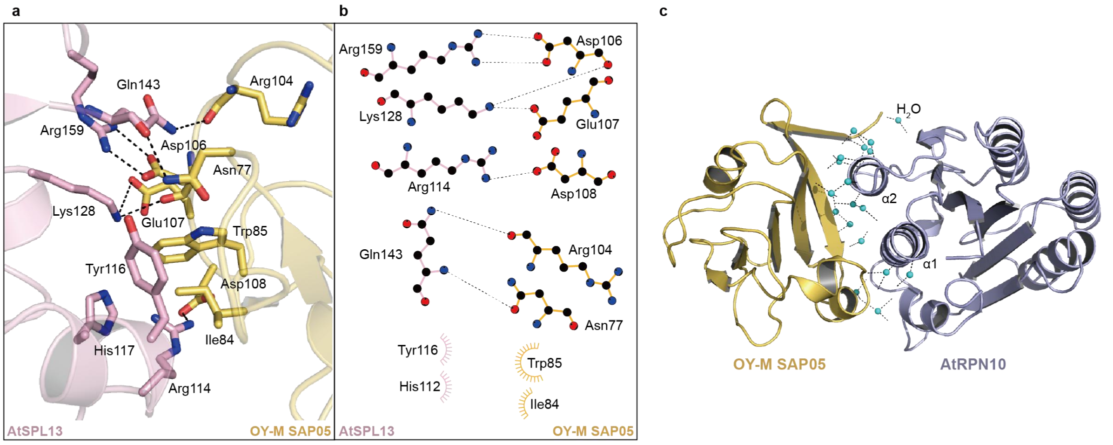

Supplementary Fig. 3 | The interactions of SAP05 with SPL13 and RPN10. a, Close-up view and ligplot diagram of the interactions of OY-M SAP05 with AtSPL13. Residues of AtSPL13 and OY-M SAP05 that are involved in the interactions are shown as lightpink and yelloworange sticks, respectively. **b,** Ligplot diagram illustrating the contacts between OY-M SAP05 and AtSPL13. **c,** Water-mediated interactions between OY-M SAP05 and AtRPN10. The black dashed lines indicate the water-mediated hydrogen bonds with SAP05 and RPN10. For clarity, the residues involved in the water-mediated interactions are not shown as sticks.

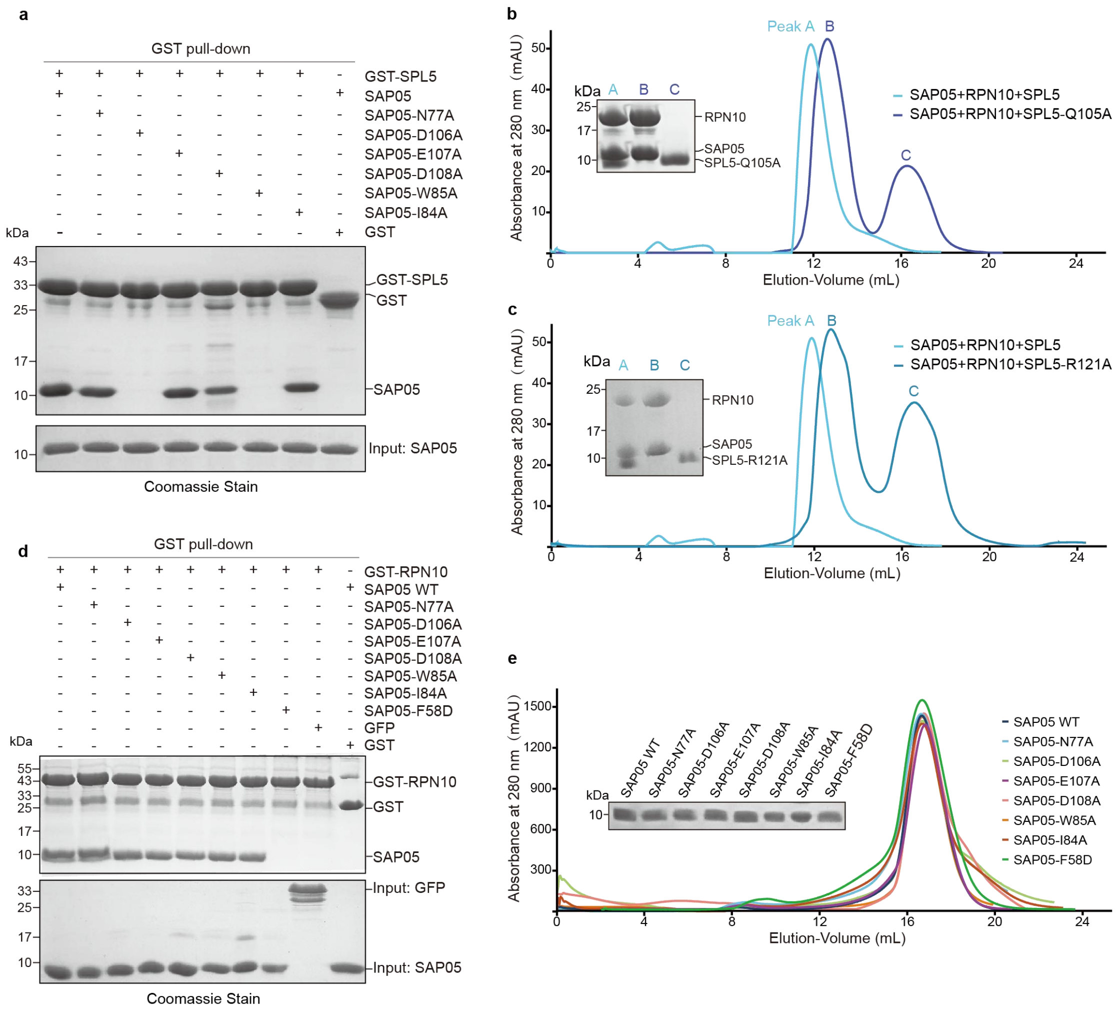

Supplementary Fig. 4 | The key residues of AtSPL5 for SAP05 binding. a, GST pull-down assay using GST-tagged AtSPL5 to pull down wild-type and mutant OY-M SAP05. **b-c,** Gel-filtration chromatography profiles of SPL5 or its mutants coeluted with SAP05-RPN10, as analyzed by Superdex 75 Increase 10/300. The left panel shows an SDS-PAGE gel of the peak fractions, stained with Coomassie blue. **d,** GST pull-down assay using GST-tagged RPN10 to pull down wild-type and TFs-binding-deficient mutant OY-M SAP05. SAP05-F58D (a mutant defective in RPN10 binding) and GFP protein are used as negative controls. **e,** Superdex 75 Increase 10/300 gel-filtration chromatography profiles of wild-type and mutant SAP05. Source data are provided as a Source Data file. Representative images, n=3.

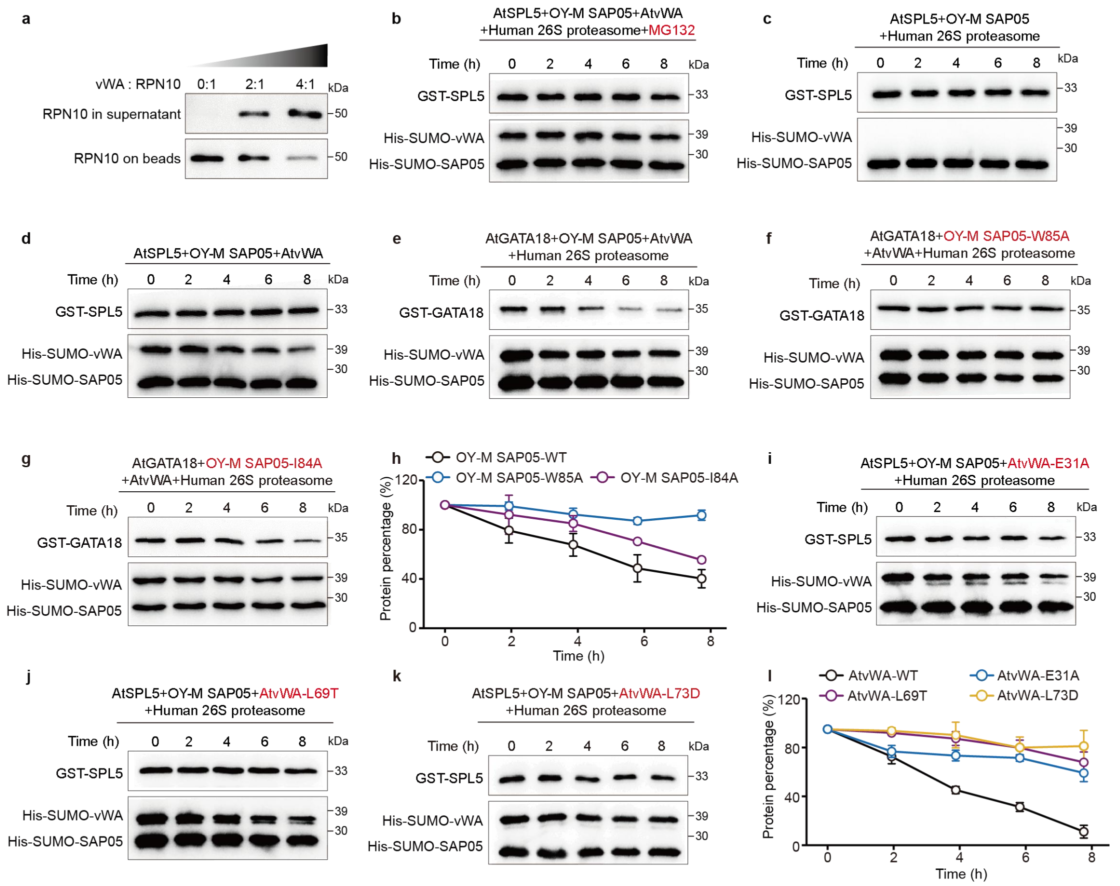

Supplementary Fig. 5 | Western blot analysis of OY-M SAP05-mediated degradation of AtSPL5. a, Western blot analysis of vWA competition assay. As the molar ratio of vWA to RPN10 increased (0:1, 2:1, 4:1), more RPN10 was competitively replaced by vWA on the purified human 26S proteasomes. **b,** Western blot analysis of OY-M SAP05-mediated degradation of AtSPL5 with proteasome inhibitor MG132. **c,** Western blot analysis of OY-M SAP05-mediated degradation of AtSPL5 in the absence of AtRPN10 vWA domain. **d,** Western blot analysis of OY-M SAP05-mediated degradation of AtSPL5 in the absence of 26S proteasome. **e,** Western blot analysis of wild-type OY-M SAP05-mediated degradation of wild-type AtGATA18. **f-g,** Western blot analysis of the degradation of AtSGATA18 in the presence of OY-M SAP05 mutants (W85A or I84A). **h,** Quantification of the percentage of retained GST-GATA18 in the degradation assay, corresponding to **e-g** (Mean $\pm$ S.E.M.; n = 3 independent experiments). **i-k,** Western blot analysis of the degradation of AtSPL5 by the AtvWA mutant (E31A, L69T or L73D). Source data are provided as a Source Data file. Representative images, n=3. **l,** Quantification of the

percentage of retained GST-SPL5 in the degradation assay, corresponding to Fig. 4a and Supplementary **i-k** (Mean ± S.E.M.; n = 3 independent experiments).

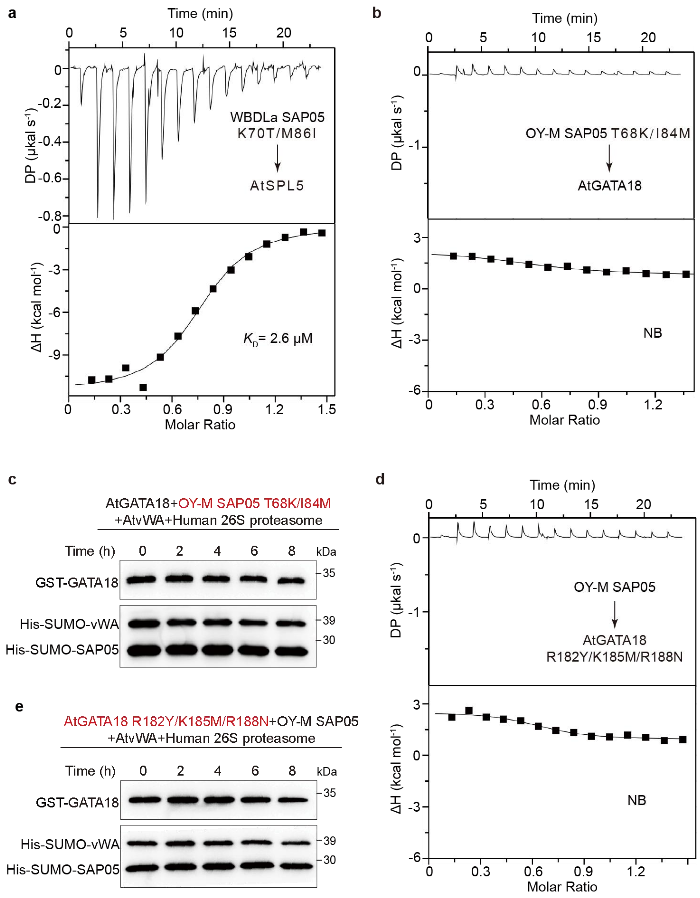

Supplementary Fig. 6 | Binding and degradation analysis of SAP05 and TFs mutations. a, ITC measurement of binding affinity of double mutant WBDLa (K70T, M86I) to AtSPL5. **b,** ITC measurement of binding affinity of double mutant OY-M SAP05 (T68K, I84M) to AtGATA18. NB, no apparent binding under our experimental conditions. **c,** Western blot analysis of mutant OY-M SAP05 (T68K, I84M)-mediated degradation of GATA18 in purified human 26S proteasomes. **d,** ITC measurement of binding affinity of OY-M SAP05 to triple mutant AtGATA18 (R182Y, K185M, R188N). NB, no apparent binding under our experimental conditions. **e,** Western blot analysis of OY-M SAP05-mediated degradation of mutant GATA18 (R182Y, K185M, R188N) in purified human 26S proteasomes. Source data are provided as a Source Data file. Representative images, n=3.

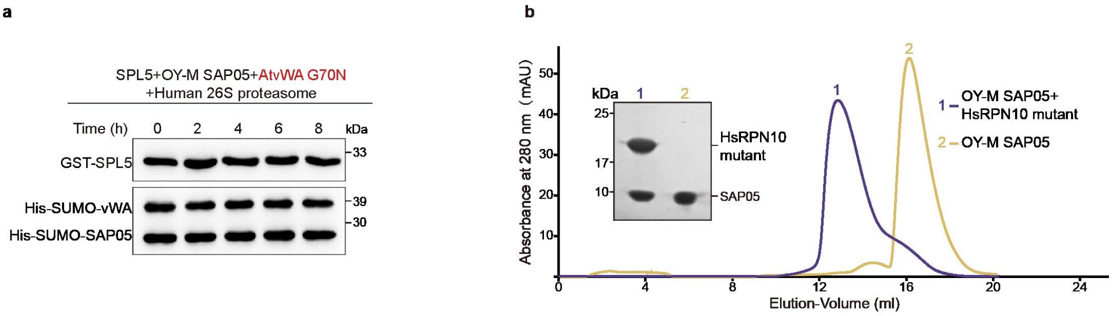

Supplementary Fig. 7 | Degradation and binding analysis of AtvWA and HsRPN10 mutations. a, Western blot analysis of OY-M SAP05-mediated degradation of SPL5 in the presence of mutant AtvWA domain (G70N) in purified human 26S proteasomes. **b,** Gel-filtration chromatography profiles of SAP05 alone and in complex with human RPN10 mutant, as analyzed by Superdex 75 Increase 10/300. The left panel shows an SDS-PAGE gel of the peak fractions, stained with Coomassie blue. Source data are provided as a Source Data file. Representative images, n=3.

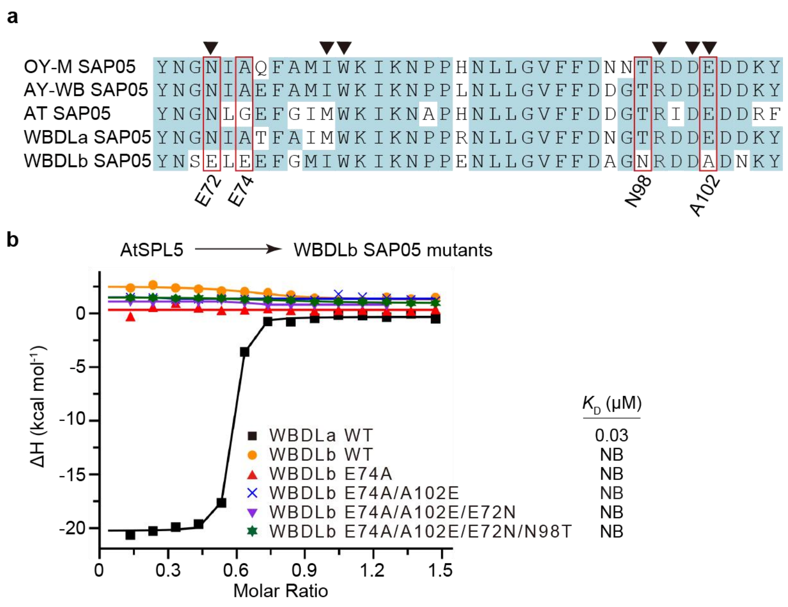

Supplementary Fig. 8 | Binding analysis of AtSPL5 with wild-type and mutant WBDLb SAP05. a, Sequence alignment of SAP05 homologs in divergent phytoplasmas. Identical residues are marked by lightblue background. The residues that directly interact with AtSPL5 are numbered and denoted by black triangles. The potential residues required for AtSPL5 binding are indicated by red frames. **b,** ITC measurements of binding affinities (K_D) of AtSPL5 to wild-type and mutant WBDLb SAP05. The ITC titration of AtSPL5 to wild-type WBDLa SAP05 was presented here as control. NB, no apparent binding under our experimental conditions.

Table S1 | All the ITC data with K_D value (mean ± SD) and corresponding N value.

**Syringe**	**Cell**	**K_D (μM)**	**N**
OY-M SAP05 WT	AtSPL5 WT	0.42 ± 0.08	0.84
OY-M SAP05 N77A	AtSPL5 WT	0.91 ± 0.51	0.79
OY-M SAP05 W85A	AtSPL5 WT	NB	
OY-M SAP05 D106A	AtSPL5 WT	NB	
OY-M SAP05 E107A	AtSPL5 WT	6.3 ± 1.29	0.74
OY-M SAP05 WT	AtSPL5 Y78A	NB	
OY-M SAP05 WT	AtSPL5 Y79A	NB	
OY-M SAP05 WT	AtSPL5 Q105A	NB	
OY-M SAP05 WT	AtSPL5 S108A	1.4 ± 0.014	0.68
OY-M SAP05 WT	AtSPL5 R121A	NB	
OY-M SAP05 WT+AtRPN10	AtSPL5 WT	0.37 ± 0.11	0.60
OY-M SAP05 WT	AtGATA18 WT	0.14 ± 0.02	0.90
OY-M SAP0 I84A	AtGATA18 WT	402 ± 31	0.52
OY-M SAP0 W85A	AtGATA18 WT	NB	
OY-M SAP05 D106A	AtGATA18 WT	4.0 ± 0.80	0.60
OY-M SAP05 E107A	AtGATA18 WT	1.3 ± 0.18	0.99
OY-M SAP05 D108A	AtGATA18 WT	5.4 ± 0.04	0.50
OY-M SAP05 WT	AtGATA18 I181A	5.0 ± 0.65	0.56
OY-M SAP05 WT	AtGATA18 R182A	4.4 ± 0.49	0.69
OY-M SAP05 WT	AtGATA18 K185A	0.58 ± 0.06	0.60
OY-M SAP05 WT	AtGATA18 R188A	0.50 ± 0.02	0.66
OY-M SAP05 WT+AtRPN10	AtGATA18 WT	0.10 ± 0.02	0.70
WBDLa SAP05 WT	AtGATA18 WT	NB	
WBDLa SAP05 M86I	AtGATA18 WT	NB	
WBDLa SAP05 K70T	AtGATA18 WT	NB	
WBDLa SAP05 K70T/M86I	AtGATA18 WT	2.7 ± 1.26	0.90
WBDLa SAP05 K70T/M86I	AtSPL5 WT	2.6 ± 1.45	0.64
OY-M SAP05 T68K/I84M	AtGATA18 WT	NB	
OY-M SAP05 WT	MqGATA18 WT	NB	
OY-M SAP05 WT	MqGATA18 Y-R/M-K/N-R	0.90 ± 0.31	0.67
OY-M SAP05 WT	AtGATA18 R182Y/K185M/R188N	NB	
AtSPL5 WT	WBDLa SAP05 WT	0.03 ± 0.002	0.63
AtSPL5 WT	WBDLb SAP05 WT	NB	
AtSPL5 WT	WBDLb SAP05 E74A	NB	
AtSPL5 WT	WBDLb SAP05 E74A/A102E	NB	
AtSPL5 WT	WBDLb SAP05 E74A/A102E/E72N	NB	
AtSPL5 WT	WBDLb SAP05 E74A/A102E/E72N/N98T	NB	
