# Supplementary material for: Molecular basis of SAP05-mediated ubiquitin-independent proteasomal degradation of transcription factors
Source: Nat Commun. 2024 Feb 8;15:1170. doi: 10.1038/s41467-024-45521-7 (PMC10850148; doi:10.1038/s41467-024-45521-7)
Supplement: Supplementary file 3 — Reporting Summary [file 41467_2024_45521_MOESM3_ESM.pdf]

nature portfolio

Corresponding author(s): Cheng Dong

Last updated by author(s): Dec 13, 2023

Reporting Summary

Nature Portfolio wishes to improve the reproducibility of the work that we publish. This form provides structure for consistency and transparency in reporting. For further information on Nature Portfolio policies, see our Editorial Policies and the Editorial Policy Checklist.

Statistics

For all statistical analyses, confirm that the following items are present in the figure legend, table legend, main text, or Methods section.

n/a	Confirmed	
[ ]	[x]	The exact sample size (n) for each experimental group/condition, given as a discrete number and unit of measurement
[ ]	[x]	A statement on whether measurements were taken from distinct samples or whether the same sample was measured repeatedly
[ ]	[x]	The statistical test(s) used AND whether they are one- or two-sided<br>*Only common tests should be described solely by name; describe more complex techniques in the Methods section.*
[x]	[ ]	A description of all covariates tested
[x]	[ ]	A description of any assumptions or corrections, such as tests of normality and adjustment for multiple comparisons
[ ]	[x]	A full description of the statistical parameters including central tendency (e.g. means) or other basic estimates (e.g. regression coefficient) AND variation (e.g. standard deviation) or associated estimates of uncertainty (e.g. confidence intervals)
[ ]	[x]	For null hypothesis testing, the test statistic (e.g. F, t, r) with confidence intervals, effect sizes, degrees of freedom and P value noted<br>*Give P values as exact values whenever suitable.*
[x]	[ ]	For Bayesian analysis, information on the choice of priors and Markov chain Monte Carlo settings
[x]	[ ]	For hierarchical and complex designs, identification of the appropriate level for tests and full reporting of outcomes
[x]	[ ]	Estimates of effect sizes (e.g. Cohen's d, Pearson's r), indicating how they were calculated

Our web collection on statistics for biologists contains articles on many of the points above.

Software and code

Policy information about availability of computer code

Data collection	SSRF BL02U and BL18U
Data analysis	XDS, PHASER in PHENIX1.11.1, COOT 0.8.7.1, PyMOL 1.7.0.1.

For manuscripts utilizing custom algorithms or software that are central to the research but not yet described in published literature, software must be made available to editors and reviewers. We strongly encourage code deposition in a community repository (e.g. GitHub). See the Nature Portfolio guidelines for submitting code & software for further information.

Data

Policy information about availability of data

All manuscripts must include a data availability statement. This statement should provide the following information, where applicable:

- Accession codes, unique identifiers, or web links for publicly available datasets
- A description of any restrictions on data availability
- For clinical datasets or third party data, please ensure that the statement adheres to our policy

Atomic coordinates for the reported structures have been deposited in the Protein Data Bank (PDB) under accession numbers 8J48 (for GATA18-SAP05), 8J49 (SPL5-SAP05), 8J4B (SPL13-SAP05) and 8J4A (SAP05-RPN10). All data needed to evaluate the conclusions in the paper are present in the paper and/or the Supplementary Information.

Research involving human participants, their data, or biological material

Policy information about studies with human participants or human data. See also policy information about sex, gender (identity/presentation), and sexual orientation and race, ethnicity and racism.

Reporting on sex and gender	N/A
Reporting on race, ethnicity, or other socially relevant groupings	N/A
Population characteristics	N/A
Recruitment	N/A
Ethics oversight	N/A

Note that full information on the approval of the study protocol must also be provided in the manuscript.

Field-specific reporting

Please select the one below that is the best fit for your research. If you are not sure, read the appropriate sections before making your selection.

[x] Life sciences [ ] Behavioural & social sciences [ ] Ecological, evolutionary & environmental sciences

For a reference copy of the document with all sections, see nature.com/documents/nr-reporting-summary-flat.pdf

Life sciences study design

All studies must disclose on these points even when the disclosure is negative.

Sample size	No statistical methods were used to predetermine sample sizes. Sample sizes n≥3. Sample size as estimated according to previous successful experience and to be large enough to obtain reproducible results.
Data exclusions	No data were excluded from analyses.
Replication	Each assay was performed at least two times. All attempts at replication were successful.
Randomization	All samples were analyzed equally with no subsampling. Therefore, there was no requirement for randomization.
Blinding	Investigators were generally not blinded as the experimental conditions required investigators to know the identity of the samples.

Reporting for specific materials, systems and methods

We require information from authors about some types of materials, experimental systems and methods used in many studies. Here, indicate whether each material, system or method listed is relevant to your study. If you are not sure if a list item applies to your research, read the appropriate section before selecting a response.

Materials & experimental systems

n/a	Involved in the study	
[ ]	[x]	Antibodies
[ ]	[x]	Eukaryotic cell lines
[x]	[ ]	Palaeontology and archaeology
[x]	[ ]	Animals and other organisms
[x]	[ ]	Clinical data
[x]	[ ]	Dual use research of concern
[x]	[ ]	Plants

Methods

n/a	Involved in the study	
[x]	[ ]	ChIP-seq
[x]	[ ]	Flow cytometry
[x]	[ ]	MRI-based neuroimaging

Antibodies

Antibodies used	Anti-His (1:1000, ZSGB-Bio, TA-02), Anti-GST (1:2000, Beyotime, AF5063), Anti-RPN10 (1:1000, Proteintech, 14899-1-AP)
Validation	All Antibodies were validated by the manufacturer. In addition, we validated that all antibodies showed the expected phenotype for a given assay.<br>The His tag is used for detection of His fusion proteins, corresponding to amino acid residues HHHHHHHH. In our experiment, HA tag

is fused to Rpn10 vWA domain or SAP05 at N-terminus. The western blot result shows HA antibody recognized HA-fused Rpn10 vWA domain or SAP05 but not the control sample.
The GST tag is used for detection of GST fusion proteins. In our experiment, GST tag is fused to SPL5 at N-terminus. The western blot result shows GST antibody recognized GST-fused SPL5.
Anti-RPN10 is used for detection of RPN10. In our experiment, the western blot result shows RPN10 antibody recognized RPN10.

Eukaryotic cell lines

Policy information about cell lines and Sex and Gender in Research

Cell line source(s)	HEK293T cells from ATCC, CRL-3216
Authentication	The HEK293T cell was authenticated by short tandem repeat (STR) profiling at Genewiz Company.
Mycoplasma contamination	The HEK293T cell was tested negtive for mycoplasma contamination.
Commonly misidentified lines (See ICLAC register)	No commonly misdentified cell lines were used in the study.

Plants

Seed stocks	N/A
Novel plant genotypes	N/A
Authentication	N/A
